# Effects on Microbiota Composition after Consumption of Quinoa Beverage Fermented by a Novel Xylose-Metabolizing *L. plantarum* Strain

**DOI:** 10.3390/nu13103318

**Published:** 2021-09-23

**Authors:** Pamela Canaviri-Paz, Elin Oscarsson, Anna Kjellström, Hanna Olsson, Chandana Jois, Åsa Håkansson

**Affiliations:** Department of Food Technology, Engineering and Nutrition, Chemical Center, Lund University, Box 124, 221 00 Lund, Sweden; elin.oscarsson@food.lth.se (E.O.); anna.kjellstrom@food.lth.se (A.K.); hanna.louise.olsson@gmail.com (H.O.); chandana.jois1@gmail.com (C.J.); asa.hakansson@food.lth.se (Å.H.)

**Keywords:** *Lactiplantibacillus plantarum* P31891, quinoa-based, fermentation, in vivo study, terminal restriction fragment length polymorphism, Next Generation Sequencing

## Abstract

Demands for novel lactic acid bacteria with potential to be used as probiotics along with healthy fermented plant-based products increase worldwide. In this study, a novel *Lactiplantibacillus plantarum* P31891 strain with enzymatic capacity to degrade tannins and ferment xylose was used as starter culture for fermentation of a quinoa-based beverage. The probiotic potential of the selected strain was evaluated in healthy volunteers. Twenty participants consumed the beverage for 14 days; microbiota changes in saliva and faecal samples were analyzed by Terminal Restriction Fragment Length Polymorphism (T-RFLP), Next Generation Sequencing (NGS) and qPCR; and gastrointestinal well-being and digestive symptoms were recorded. The results indicated that the consumption of the beverage with *Lactiplantibacillus plantarum* P31891 in a probiotic dose (10^12^ CFU/mL) increased the number of *Lactobacillus* in the feces but not in saliva. Overall, the bacterial community did not seem to be influenced by the bacterium or by the beverage, as expressed by the diversity indexes, but specific genera were affected, as reflected in changes in amplicon sequence variants. Consequently, *Lactiplantibacillus plantarum* P31891 showed potential to be categorized as a probiotic strain in the fermented quinoa-based beverage.

## 1. Introduction

The consumption of beverages and foods that contain probiotic microorganisms has in recent years shown tremendous growth worldwide. Although fermented dairy products are currently the most common food carrier and generally good matrices to deliver probiotics to humans, an increasing number of non-dairy food matrices exhibit potential to be used. Due to a constantly increasing consumer demand of plant-based milk alternatives for sustainability, health-related, lifestyle and dietary reasons, various plants have been used to produce non-dairy options. This has resulted in an abundance of products based on nuts, seeds or beans, but the choice of substrate often limits people suffering from food allergies, and very few of the products on the market are carrier of probiotic bacteria.

Quinoa (*Chenopodium quinoa* Willd.) is a native plant in the Andean region of South America that has attracted global growing interest and expanded cultivation even at European latitudes, where the number of producer countries are rapidly increasing [1]. The nutritional value of quinoa grains is high, with a protein quality similar to milk [2] while being gluten free. Quinoa also has a relatively high amount of dietary fibers, facilitating digestion [3]. Due to the substantial nutritional content, quinoa is an interesting food base for functional foods. However, quinoa is in addition known for some anti-nutritional aspects such as content of saponins and phytic acid that can be reduced by fermentation. The process can furthermore increase bioavailability of several compounds [4,5], and fermented foods have been associated with health benefits by, for example, reducing the risk of diseases such as type 2 diabetes [6,7]. Traditionally, fermentation has been used as a food preserving technique for thousands of years, and both the process and the products have recently attracted scientific interest due to claimed beneficial effects. The fermentation process is usually performed by the diverse group of bacteria called lactic acid bacteria (LAB) including, for example, the family *Lactobacillaceae*. A properly fermented product is microbiologically safer and has a longer shelf-life compared to an unfermented one. However, induced fermentation relies on the efficacy of the starter culture, and it is important to use a culture that can outcompete the native microbiota of the substrate used. Quinoa has been related to inadequate hygiene after spontaneous fermentation or via backslopping, as the native microbiota of quinoa grains comprises opportunistic pathogens [8]. 

The composition of our gut microbiota and dietary factors are significant for human health, and the use of probiotics and prebiotics can play an important role in its maintenance [9,10]. Probiotic bacteria commonly belong to lactic acid bacteria and can, therefore, be found in spontaneously fermented products. As probiotics have been reported to, e.g., strengthen the intestinal barrier function, to compete with intestinal pathogens and to produce health promoting substances and stimulate the immune system [11,12,13], the use of probiotic strains as starter cultures can contribute to additional health benefits in fermented products. In this study, the strain *Lactiplantibacillus plantarum* P31891 was used as starter culture due to its ability to degrade tannins, to ferment xylose [14] and for being a safe organism to consume [15]. According to the definition, probiotic organisms should be viable and consumed in adequate amounts in order to influence the composition of the intestinal microbiota. Lactic acid bacteria as starter culture in a quinoa-based fermented drink has previously been found to survive transit through the gastrointestinal tract, indicating that fermented quinoa might be a suitable medium for probiotic bacteria [16].

The aim of this study was to evaluate the probiotic potential of a quinoa-based fermented drink using the bacterium *Lactiplantibacillus plantarum* P31891 as a starter culture, with focuses on changes in saliva and faecal microbiota composition after 14 days of daily consumption by healthy volunteers. 

## 2. Materials and Methods

### 2.1. Beverage Development and Evaluation of Hygiene Quality

#### 2.1.1. Quinoa-Based Fermented Drink

White quinoa grains (*Chenopodium quinoa* Willd.) (Sålta Kvarn AB, Stockolm, Sweden) imported from Bolivia were acquired from a local supermarket in Lund, Sweden. A quinoa-based fermented drink was produced in a pilot scale at the Department of Food Technology, Engineering and Nutrition, Lund University. Elimination of possible impurities on quinoa grains was performed by submerging the grains in water 1:3 (*w*/*v*) twice, under sporadic agitation for 15 min each. The cleaning continued under running tap water until the discarded water became clear and foamless. The clean quinoa grains were dried at 195 °C with constant agitation on a stove (Elektro Helios, Stockholm, Sweden), followed by 20 min toasting at 145 °C. Sterile tap water, autoclaved at 121 °C for 15 min and cooled down to 4 °C, was mixed with the toasted quinoa grains in a proportion of 1:8 (*w*/*v*). The mixture was homogenized using a blender (Electrolux, Great blending TruFlowTM blades, ESB5400BK, Stockholm, Sweden) for 15 min. Subsequently, the mixture was filtrated through a cheese cloth, collected and distributed in one liter (302.135.52, IKEA, Malmö, Sweden) or half liter (203.244.72, IKEA, Malmö, Sweden) glass bottles with caps, which were filled to the maximum capacity after inoculation and sealed hermetically, minimizing the presence of oxygen. The inoculum concentration was measured spectrophotometrically at 610 nm. The quinoa beverage was inoculated with 1.6 × 10^9^ CFU of *Lactiplantibacillus plantarum* P31891 per liter beverage. The quinoa-based drink was incubated at 30 °C for 48 h with sporadic agitation and thereafter stored at 4 °C before distribution. 

#### 2.1.2. pH and Microbial Analysis

Changes in pH and plate count were assessed for hygiene evaluation of the drink before fermentation (0 time), after fermentation (48 h) and at the seventh and fourteenth days of storage in 4 °C. The pH was measured using a Methohm 744 pHmeter (Metholhm, Ltd., Herisau, Switzerland) previous calibrated according to the manufacturer’s recommendations. For viable count, a conventional dilution procedure was conducted, and samples from the selected dilutions were spread plated on Violet Red Bile Dextrose agar (VRBD, Merck, Germany) incubated aerobically at 37 °C during 24 h for enumeration of *Enterobacteriaceae* and on Rogosa agar (Oxoid) incubated anaerobically (Gas Pak Anaerobic system, BBl, Becton Dickinson and company, Franklin Lakes, NJ, USA) at 37 °C for 72 h for lactobacilli count.

### 2.2. In Vivo Study

Volunteers for participation in the study were recruited during November and December (2019) in Sweden. Inclusion criteria were healthy individuals equal or over 18 years old who had not been under medical treatment in the past year or consumed antibiotics 6 months before their participation in the study. The participants did not suffer from gastrointestinal disorders or symptoms such as irritable bowel syndrome, inflammatory bowel disease, constipation or diarrhea at inclusion. The number of participants was determined based on the power analysis (PS Power and sample Size Program), version 3.0, William D. Dupont and Walton D. Plummer, to reach a power of 80%. Participants (13 females and 7 males) were enrolled after signing the consents to participate in the study according to the Declaration of Helsinki. The study was approved by the Swedish Ethical Review Authority (Dnr: 2019-01302) and is registered in ClinicalTrials.gov (NCT04280731).

#### 2.2.1. Protocol

The study continued for 30 days. The participants were subjected to two weeks (14 d) of a wash out period prior to consumption. The participants were told to not consume any commercial probiotic products or fermented products during the study. During the consecutive two weeks (14 d), the participants consumed daily 2.5 dL of the fermented quinoa beverage that they were instructed to keep in the fridge (approx. 4 °C) at home. Along with the drink, the participants received an exploratory questionnaire and pre-labelled tubes for collecting saliva (50 mL, VWR) and stool samples (Feces tube 76 × 20 mm, SARSTED). The participants recorded gastrointestinal well-being and digestive symptoms in a questionnaire during the study.

#### 2.2.2. Saliva and Stool Samples Collection

Saliva and stool samples were collected by the participants at two occasions: one day (24 h) before starting consumption of the fermented quinoa beverage and one day (24 h) after the last day of consumption. After collection, the samples were stored at refrigeration temperature (faecal and saliva samples for qPCR analysis) and in −20 °C (faecal and saliva samples for T-RFLP and NGS analysis) by the participants until delivery to the Department of Food Technology Engineering and Nutrition, Lund University. The samples were further stored at 4 °C or −80 °C until being analyzed using the methods mentioned.

### 2.3. Qualitative and Quantitative Assay of In Vivo Samples

#### 2.3.1. DNA Extraction

Saliva and faecal samples stored at 4 °C were treated immediately after arrival to Department of Food Technology Engineering and Nutrition. Aliquots of 100 µL of saliva (n = 3, per participant) were transferred into 5 mL Man Rogosa and Shape Broth (MRS-Broth, Merck, Germany) incubated at 37 °C anaerobically for 24 h. Faecal samples were weighed (1 g) and diluted into 9 mL of sterile peptone water and vortexed for 1 min. Aliquots of 1 mL of faecal sample (n = 3, per participant) were transferred into 5 mL of MRS broth. Incubation was followed as previously described [17]. After incubation, the samples were centrifuged at 3000× *g* for 5 min. After centrifugation, 500 µL of the supernatant were withdrawn and pulled together per participant and centrifuged at 20.8× *g* for 3 min (Thermo Scientific, Heraeus Pico 21, Hamburg, Germany). The pellets were cleaned with 500 µL of 0.85% NaCl (g/L) sterile solution followed by a second clean using 500 µL of sterile Milli-Q water. The pellets were suspended in 500 µL of sterile Milli-Q water before bead beating using an Eppedorf Mixer 5432 (Eppendorf, Hamburg, Germany) for 45 min at 4 °C. The DNA was separated by centrifugation (20.8× *g*, 1 min) and used as templated in the subsequent polymerase chain reaction (PCR).

DNA was extracted from frozen stool samples according to the method described by Karlsson et al. [18]. Briefly, 50 mg of stool sample was weighed in 1.5 mL tubes containing glass beads (between 10 to 12, 2 mm diameter, previously UV sterilized) using an analytical weigh (model TA302-Traveler, Switzerland), and 500 µL of sterile PBS (Phosphate Buffered Saline; pH 7.3 ± 0.2 at 25 °C) was added. The samples were incubated during 10 min at room temperature and vortexed for 1 min followed by 45 min of bead beating at 4 °C on an Eppendorf Mixer (model 5432, Eppendorf, Hamburg, Germany), after which they were vortexed for 1 min and centrifuged (3000× *g*, 30 s) to sediment the pellet. The amount of 200 µL of the supernatant was collected for DNA extraction using the DNA tissue kit (Qiagen bioinformatics, Aarhus, Denmark) and EZ1 advanced XL BioRobot (tissue kit and card; Qiagen). The samples were store at 4 °C until further analyses.

DNA was extracted from 200 µL of frozen saliva samples after thawing on ice without previous treatment. The DNA was extracted as previously described, but the bead beating procedure was excluded. The samples were stored at 4 °C until further analyses.

#### 2.3.2. Terminal Restriction Fragment Length Polymorphism Assay

The microbial communities from the saliva and faecal samples were characterized by amplifying the 16S rRNA genes by the pair primers listed in Table 1. The PCR reactions were performed in six replicates. The Shannon and Simpsons diversity index (DI) was calculated from the peak area (40–600 bp) obtained per sample in Excel (Microsoft, 2010), according to the method described by Oscarsson et al. [19].

#### 2.3.3. Next Generation Sequencing

The variable region V3-V4 was amplified using the primers (Eurofins Genomics, Ebersberg, Germany) in Table 1 according to the 16S Metagenomic Sequencing Library Preparation protocol. The thermal cycling was performed in an Eppendorf MasterCycler (Eppendorf, Hamburg, Germany), and the resulting fragments were 550 bp. AMPure XP beads (Agencourt, Beckman coulter genomics, Brea, CA, USA) were used for purification of the amplified PCR products. A second PCR reaction was performed in order to attach indexes (Nextera XT index kit, Illumina, San Diego, CA, USA) to the fragments, and the PCR products were purified once more. The concentration of the resulting DNA fragments was determined by using Qubit4.0 Fluorometer (Thermo Fisher Scientific, MAN0017210, Gothenborg, Sweden), after which the samples were combined in equimolar ratios to a final concentration of 6 pM. The fragments were loaded to an Illumina MiSeq system (Illumina, San Diego, CA, USA) and sequenced using MiSeq reagent kit v3 (Illumina Inc., San Diego, CA, USA) with a read length of 2 × 300 bp paired-end sequencing according to the manufacturer’s instructions.

#### 2.3.4. Quantitative Real-Time PCR

The amount of DNA originating from bacteria belonging to the former genus *Lactobacillus* was determined in faecal and saliva samples using a q-PCR assay following the procedures previously described by Karlsson et al. [17]. Briefly, each reaction contained 10 µL 2xRotor-Gene SYBR Green PCR Master Mix (Qiagen) together with 0.5 µmol/L of the primers listed in Table 1, 2 µL DNA template and RNAse-free water, resulting in a final volume of 20 µL. All samples, standards and negative controls were run in triplicates in a Rotor-Gene Q (Qiagen). The thermal cycling started with 95 °C for 5 min followed by 40 cycles where the DNA was denatured at 95 °C for 5 s, annealed and elongated at 60 °C for 20 s. The absolute amount of DNA belonging to *Lactobacillus* was determined based on calculations using standard curves with known DNA concentrations using Rotor-Gene Q Series Software 1.7 (Qiagen), R^2^ > 0.998. The detection limit was 10^2^ genes/reaction. Cloned PCR products from *Lactiplantibacillus plantarum* 299v was used for building the standard curve.

In order to confirm viability of *Lactobacillus* spp. in faecal and saliva samples after consumption, viable count was performed on Rogosa agar (Oxoid) after serial dilution in sterile bacteriological peptone water (NaCl, Merck, 8.5 g/L; Bacteriological peptone, Oxoid, 1g/L), and the samples were incubated anaerobically (Gas Pack Anaerobic system, BBI, Becton Dickinson and company, Franklin Lakes, NJ, USA) at 37 °C for 72 h.

### 2.4. Statistical Analysis

SigmaPlot version 14.0 (SYSTAT Software, Point Richmond, CA, USA) software was used for the statistical analyses. Changes in pH and viable cell counts were evaluated by Kruskal–Wallis one-way analysis of variance (ANOVA) on ranks or a Mann–Whitney ranks sum test, when appropriate. T-RFLP and q-PCR data were analysed by Wilcoxon signed rank test, and the results are expressed as median and interquartile range (IQR; 25–75%). For viable count of lactobacilli after consumption, Excel 2010 was used, and the results were expressed as mean (SD). NGS statistical analyses were calculated in R version 3.6.3 excluding all sequences not identified as bacteria, such as chloroplasts or mitochondria, prior analysis. The differences in Alpha diversity (Observed, Chao1, Shannon and Simpson) over time were calculated with the Wilcoxon test for pairwise comparison. Beta diversity was calculated using the weighted and unweighted unifrac, Jaccard and Bray-Curtis dissimilarity and was compared using the Adonis test. The differences between timepoints regarding relative abundance of phyla, family and genus level were computed with the Wilcoxon rank test, and DeSeq2 was used for calculating Log2Fold change. The data were normalized using total sum scaling prior calculation of the relative abundance test. *p*-values ≤ 0.05 were considered significant.

## 3. Results

### 3.1. Beverage Development and Hygiene Evaluation

#### pH and Microbial Analysis

The efficiency of the fermentation and the safety of the quinoa based fermented drink were evaluated by monitoring pH and plate culturing during the fermentation. A significant decrease in pH was observed after 48 h of fermentation (*p* < 0.001; Table 2) with a slightly continuous variation during storage time. The fermentation was not dependent on the volume of substrate used for the process, and no significant differences of pH or viable count were detected between volumes (Table 2). The presence–absence of *Enterobacteriaceae* before and after fermentation was investigated in order to validate the hygiene quality of the beverage. The decrease in viable *Enterobacteriaceae* cells was statistically significant (*p* = 0.001) at 48 h of fermentation and remained stable below the limit of detection during the storage time (*p* = 0.017) (Table 2). The increase in bacterium *L. plantarum* P31891 was statistically significant at 48 h (*p*-values = 0.001) and remained constant during the storage time (Table 2).

### 3.2. In Vivo Study

#### Consumer Experience

Among the participants, one of the volunteers interrupted the participation in the study approximately 1 week after start but provided samples that were included in the analyses. In total, 19 volunteers completed the research study, and 17 submitted the questionnaire. Eighty percent of the participants had consumed commercial probiotic products or fermented foods prior to enrolment. During the study, 59% reported a change in stool behavior, and 29% described increased formation of gas, while 12% reported a reduced amount, and 59% did not experience any difference. Furthermore, 35% of the volunteers experienced stomach pain during the study while 65% did not.

### 3.3. Qualitative and Quantitative Assay of In Vivo Samples

#### 3.3.1. Alpha and Beta Diversity

The alpha diversity calculated based on the T-RFLP analysis as the Shannon and Simpson diversity index did not change after consumption of the fermented quinoa-based drink (saliva *p* = 0.701; *p* = 0.105 and feces *p* = 0.409; *p* = 0.294, respectively) (Table 3).

**Figure 1:**
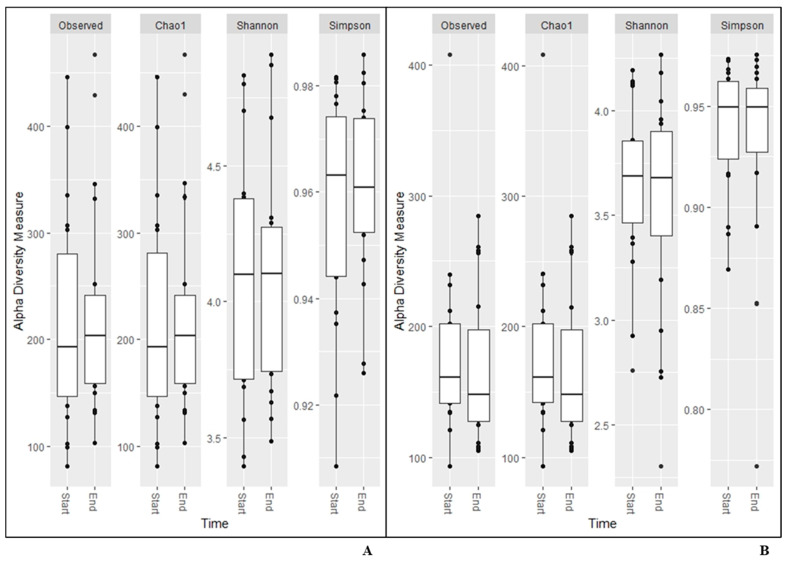
Alpha diversity measurements for saliva (**A**), and faecal (**B**) samples.

The same results were found for alpha diversity for any of the measurements calculated based on data from the NGS analysis (Figure 1). The beta diversity remained constant during the study (unweighted unifrac (*p* = 1.0; *p* = 1.0); Bray–Curtis (*p* = 1.0; *p* = 1.0); Jaccard (*p* = 1; *p* = 1), and weighted unifrac (*p* = 0.990; *p* = 0.986), respectively.

#### 3.3.2. Relative Abundance

The relative composition of different phyla can be observed in Table 4 for both saliva and faecal samples. In the saliva samples, the most occurring phyla was Bacteroidetes and Firmicutes, followed by Proteobacteria. In the faecal samples, Firmicutes had the highest relative abundance, followed by Bacteroidetes (Table 4). There was no change in relative abundance on phyla level over the study period, however, both in saliva and in faecal samples, an increase in the family *Lactobacillaceae* could be observed (*p* < 0.01 and *p* < 0.0001, respectively) (Table 5 and Table 6).

**Figure 2:**
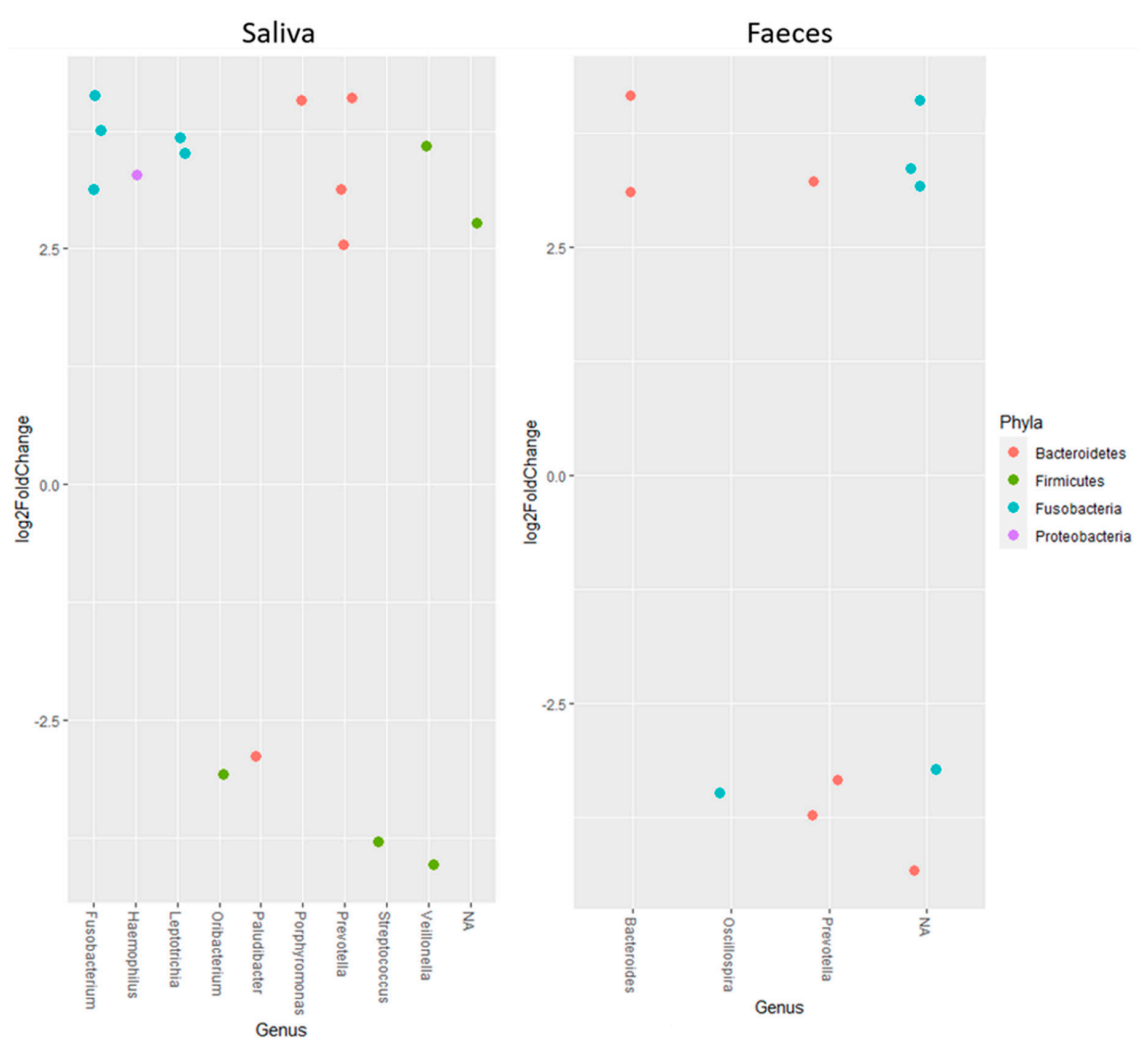
Log2FoldChange calculated as start vs. end for amplicon sequence variants belonging to different genera. All results in the figure are significant with *p* < 0.001. Positive changes indicate an increase over time, while negative values show a decrease.

Studying the change in abundance of specific ASVs over time, it was found that ASVs belonging to *Fusobacterium*, *Haemophilus*, *Leptotrichia*, *Porphyromonas* and *Prevotella* increased, meanwhile *Ornibacterium*, *Paludibacter* and *Streptococcus* decreased in saliva samples (Figure 2). For *Veillonella*, one ASV increased but another decreased. In faecal samples, ASVs belonging to *Bacteroides* increased and *Oscillospira* decreased over time, whereas one ASV of *Prevotella* increased and two decreased (Figure 2). 

#### 3.3.3. Absolute Amount of Lactobacillus

The absolute number of lactobacilli in saliva samples was not significantly different over time (Table 7). Contrary to the saliva samples, the lactobacilli content was higher in faecal samples at the end of the study, compared to the initial values (Table 7).

The viable counts of lactobacilli in saliva and faecal samples after consumption of the fermented quinoa beverage were 4.90 ± 0.52 and 7.96 ± 0.57 (mean (SD)), respectively, and growth were found for all individuals.

## 4. Discussion

The present clinical trial investigated changes in saliva and faecal microbial composition after 14 days of consumption of a quinoa-based beverage fermented with a potential probiotic species, *Lactiplantibacillus plantarum* P31891, previously isolated from quinoa grains [14]. When probiotic bacteria actively participate in the fermentation process of a product, the composition of the substrate to which they will be added, in relation to the media they originate from, is of high importance for the process outcome. *Lactiplantibacillus plantarum* P31891 was selected for its specific functional properties [14] and reached a viable population concentration of log 12 CFU/mL, which is highly above the limit of acceptability for probiotic products [22,23].

A bacterium should fulfil different criteria in order to be considered a probiotic [24], with the main aim to contribute to a healthy gut microbiota. In the present study, the concentration of *Lactobacillus* spp. increased significantly in faecal samples after consumption of the fermented beverage, indicating that the bacteria may be able to survive the harsh conditions in the gastrointestinal tract (Table 7). Previous findings of significant higher concentrations of the *Lactobacillus* genus by intake of probiotic products are also consistent with our results [25]. Even if the saliva samples did not show a similar increase over time, it does not necessarily mean that the strain did not colonize the oral cavity. 

In the present study, the results from the alpha and beta diversity analyses showed no overall change diversity indices in either saliva or faecal samples after consumption. Those results suggest that the human microbiota in healthy individuals is relatively stable and is not affected by intake of the probiotic bacteria or the fiber-rich quinoa. Previously, it has been shown that a diet rich in fiber does not change the alpha-diversity [17]. The human microbiota is generally considered adult like at three years of age and have only minor fluctuations over time [20,21]. Overall, the saliva had higher diversity than the faecal samples (Figure 1), which was also reflected in the relative abundance of phyla (Table 4). Higher alpha diversity has been detected and not detected in saliva after intake of probiotic products in previous studies by Dassi et al. [26], which partly contrasts with our results. However, the authors also found that the probiotics only had small changes on relative abundance and taxonomic distribution, even if they were able to re-identify the species from the probiotic product in saliva samples [27]. On the other hand, it has also been shown that less than 50% of the participants had the probiotic bacteria present in the saliva samples after three weeks of probiotic intervention [16]. In the saliva samples, we found higher levels of the family *Lactobacillaceae* after two weeks of consumption of the fermented quinoa drink, showing a possible persistence of the genus (Table 5). Although we did not find an increase in absolute numbers of the genus *Lactobacillus* after consumption, we observed detectable levels of *Lactobacillus* spp. in all participants at the end of the study (Table 7). Furthermore, ASVs belonging to the genus *Prevotella* increased in abundance over the study period in the saliva samples. *Prevotella* is commonly seen to increase in the gut microbiota after intake of a fiber-rich diet [28,29] and might, therefore, increase as a consequence of the consumption of quinoa [3]. In saliva, ASVs of the genera *Fusobacterium*, *Haemophilus*, *Leptotrichia*, *Porphyromonas*, *Ornibacterium*, *Paludibacter*, *Streptococcus* and *Veillonella*, constituting genera of the oral microbiome, were also found to be affected [30,31] (Figure 2). Data of compositional changes of the human salivary microbiota by dietary factors are limited, but Hansen et al. [30] did find intake of dietary components such as fibers and fatty acids to be associated with bacterial diversity, community structure and relative abundance of species-level operational taxonomic units [30]. If the changes found after consumption of the fermented quinoa-based drink depend on the potential probiotic strain, the nutritional content of quinoa grains or a combination thereof need to be further evaluated. 

Compared to the saliva samples, the faecal microbiota was relatively stable over time. No changes were found in diversity or relative abundance of specific bacteria except an increase in the family *Lactobacillaceae* (Table 6). Furthermore, only three identified genera seemed to change in ASV abundance over time (*Bacteroides*, *Oscillospira* and *Prevotella*) (Figure 2) compared to nine in the saliva samples. ASVs belonging to *Bacteroides* seemed to increase, which has mostly been associated with consumption of animal products [29], particularly with high contents of fat and protein [28]. Discrepancies against this generalization have been shown and discussed by others, where plant-based diets high in proteins and fibers also increased the abundance of *Bacteroides* spp. [32,33,34]. Quinoa has both high protein and fiber values [2,3] and might, therefore, influence the increase in *Bacteroides*. After consumption of the fermented quinoa-based drink, we also found a significant increase in absolute numbers of *Lactobacillus* spp. in faecal samples (Table 7). The finding should be regarded as beneficial and except for indicating a possible survivability of the administrated strain, the increase can also be associated with the high content of polyphenols in quinoa grains, which has previously been shown to positively affect lactobacilli proliferation [35]. 

With an increased intake of fiber and/or when starting probiotic consumption, individuals may initially experience a mild increase in gas production, bloating and some digestive problems, which normally subsides after a few weeks of regular intake. In the present study, the volunteers participating experienced the effects after intake of the quinoa-based fermented drink differently. According to the questionnaire, 35% perceived minor stomach pains during the study, meanwhile, 65% did not have any discomfort. None of the participants experienced any increase in bloating but instead experienced a reduction in bloating of 11% was actually reported.

## 5. Conclusions

In conclusion, the consumption of the quinoa based fermented drink with *Lactiplantibacillus plantarum* P31891 did increase the amount of *Lactobacillus* spp. in the faecal samples and changed the Log2Fold change of specific ASVs in both saliva and faecal samples, especially ASVs belonging to genera associated with intake of a plant-based diet. As the diversity index was not affected by the intake, the potential probiotic strain seems to enhance the intestinal *Lactobacillus* population without altering the balanced indigenous microecology of the healthy participants. 

## 6. Patents

Deposited data under the Budapest Treaty in the Belgian Coordinated Collection of Microoorganisms-BCCM. Laboratorium voor Microbiologie-Bacterienverzameling-LMG, patent collection. The following accension number has been assigned: *L. plantarum* LMG P-31891.

## Figures and Tables

**Table 1:** Primers used for amplifications.

Method	Objective	Primers	Primer Length	Ref
T-RFLP	16S rRNA genes	FAM-ENV-1	(5′-AGA GTT TGA TII TGG CTC AG-3′)	[20]
		ENV-2	(5′-CGG ITA CCT TGT TAC GAC TT-3′)	
NGS	16S rRNA genes (V3-V4)	341F	(5′-TCG TCG GCA GCG TCA GAT GTG TAT AAG AGA CAG CCT ACG GGN GGC WGC AG-3′)	[21]
		805R	(5′-GTC TCG TGG GCT CGG AGA TGT GTA TAA GAG ACA GGA CTA CHV GGG TAT CTA ATC C-3′)	
q-PCR	Genus *Lactobacillus*	Lact-F	(5′-AGC AGT AGG GAA TCT TCC A-3′)	[17]
		Lact-R	(5′-CAC CGC TAC ACA TGG AG-3′)	

**Table 2:** pH, *Enterobacteriaceae* and lactobacilli count in the fermented quinoa-based beverage.

Volume	Time	pH		Log_10_ CFU/mL			
				*Enterobacteriaceae*		Lactobacilli	
		Median	IQR	Median	IQR	Median	IQR
1 L (n = 6)	0 h	6.36 ^a^	6.31–6.40	2.9 ^a^	2.7–3.3	<1 ^a^	<1–<1
	48 h	4.03 ^b^	3.96–4.05	<1 ^b^	<1–<1	12.1 ^b^	12.1–12.2
	7 d	3.86 ^b^	3.83–3.96	<1 ^b^	<1–<1	12.2 ^b^	12.1–12.2
	14 d	3.98 ^b^	3.95–4.03	<1 ^b^	<1–<1	11.9 ^b^	11.8–12.1
0.5 L (n = 6)	0 h	6.41 ^a^	6.37–6.41	3.1 ^a^	2.7–3.2	<1 ^a^	<1–<1
	48 h	4.04 ^b^	3.98–4.05	<1 ^b^	<1–<1	12.2 ^b^	12.1–12.3
	7 d	3.88 ^b^	3.80–3.92	<1 ^b^	<1–<1	12.3 ^b^	12.1–12.5
	14 d	3.91 ^b^	3.86–3.96	<1 ^b^	<1–<1	12.1 ^b^	12.1–12.1

^a,b^ Median values within columns with unequal superscript letters were significantly different (*p*-values ≤ 0.05).

**Table 3:** Microbial community profile expressed as diversity index (DI) in in vivo samples (n = 20) before and after intake of the fermented quinoa-based drink.

Diversity Index (DI)	Saliva				Faeces			
	Start		End		Start		End	
	Median	IQR	Median	IQR	Median	IQR	Media	IQR
Shannon	2.37	2.10–2.55	2.31	1.91–2.71	2.63	1.46–3.03	2.67	2.47–2.95
Simpson	0.84	0.79–0.87	0.82	0.70–0.88	0.88	0.67–0.92	0.88	0.84–0.92

**Table 4:** Relative abundance of bacterial phyla in saliva and faecal samples expressed as median and interquartile range (MD ± IQR) at the start and end of the study.

Bacterial Phyla Relative Abundance					
	Start (%)		End (%)		*p* (Start–End)
	Median	IQR	Median	IQR	
**Saliva**					
Firmicutes	31.8	24.7–39.8	28.8	22.6–45.4	0.664
Bacteroidetes	33.0	23.2–38.7	32.7	26.0–39.0	0.908
Fusobacteria	9.0	6.6–13.4	8.2	6.5–10.8	0.707
Actinobacteria	0.6	0.3–1.2	0.6	0.3–0.9	0.583
Proteobacteria	17.8	17.8–28.0	21.3	15.5–32.6	0.506
**Faeces**					
Firmicutes	49.5	40.6–56.4	51.6	40.0–58.0	0.883
Bacteroidetes	45.2	33.5–51.3	41.6	38.0–54.3	0.758
Actinobacteria	0.14	0.08–0.3	0.13	0.07–0.2	0.639
Proteobacteria	1.0	0.6–2.0	1.1	0.5–2.0	0.925
Verrucomicrobia	0.1	0.0–2.1	0.3	0.0–2.6	0.740

**Table 5:** Relative abundance of families and genera in saliva samples expressed as median and interquartile range (MD and IQR) at the start and end of the study.

Saliva		Start (%)		End (%)		*p* (Start–End)
Family		Median	IQR	Median	IQR	
	*Campylobacteraceae*	1.49	0.81–2.64	1.18	0.70–2.18	0.418
	*Carnobacteriaceae*	0.78	0.49–1.32	0.86	0.60–1.16	0.840
	*Flavobacteriaceae*	0.93	0.48–2.34	1.20	0.50–2.97	0.931
	*Fusobacteriaceae*	5.04	2.52–7.39	4.87	3.18–6.41	0.644
	*Gemellaceae*	0.54	0.31–0.75	0.63	0.33–0.96	0.525
	*Lachnospiraceae*	1.88	1.36–3.39	1.42	0.92–2.17	0.065
	*Lactobacillaceae*	0.00	0.00–0.00	0.02	0.00–0.09	0.003
	*Leptotrichiaceae*	3.62	2.13–5.94	3.46	2.25–5.53	0.863
	*Neisseriaceae*	7.67	2.91–13.8	6.64	4.52–11.2	0.954
	*Pasteurellaceae*	8.60	6.06–11.9	10.85	8.07–17.0	0.212
	*Peptostreptococcaceae*	0.53	0.09–0.92	0.24	0.14–0.63	0.726
	*Porphyromonadaceae*	7.25	2.53–9.02	6.62	3.83–9.91	1.00
	*Prevotellaceae*	18.5	12.8–25.8	20.10	10.5–26.7	0.885
	*Streptococcaceae*	6.97	4.85–9.68	5.19	4.27–11.67	0.931
	*Veillonellaceae*	17.9	14.4–26.4	17.84	15.4–23.3	0.954
**Genus**						
	*Aggregatibacter*	0.57	0.30–0.79	0.59	0.23–0.92	0.804
	*Campylobacter*	1.49	0.81–2.64	1.18	0.70–2.18	0.418
	*Capnocytophaga*	0.93	0.48–2.34	1.20	0.50–2.97	0.931
	*Fusobacterium*	5.04	2.5–7.39	4.87	3.18–6.40	0.644
	*Granulicatella*	0.78	0.49–1.32	0.86	0.60–1.16	0.84
	*Haemophilus*	7.23	5.59–10.0	9.12	5.17–14.5	0.297
	*Leptotrichia*	2.64	1.95–4.86	2.95	1.57–5.33	0.954
	*Megasphaera*	1.12	0.35–2.19	0.49	0.22–1.38	0.181
	*Neisseria*	6.78	2.50–13.2	5.63	3.98–10.7	1.00
	*Oribacterium*	0.93	0.67–2.06	0.72	0.42–0.94	0.061
	*Porphyromonas*	6.60	2.28–8.73	6.07	3.43–9.56	0.954
	*Prevotella*	18.48	12.8–25.8	20.1	10.5–26.7	0.885
	*Selenomonas*	0.56	0.34–1.48	0.50	0.32–1.11	0.544
	*Streptococcus*	6.97	4.85–9.68	5.19	4.27–11.7	0.931
	*Veillonella*	14.09	12.4–21.6	16.7	13.4–19.9	0.954

**Table 6:** Relative abundance of families and genera in faecal samples expressed as median and interquartile range (MD±IQR) at the start and end of the study.

Faecal		Start (%)		End (%)		*p* (Start–End)
Family		Median	IQR	Median	IQR	
	*Bacteroidaceae*	18.75	14.2–30.8	21.0	15.3–36.1	0.494
	*Lachnospiraceae*	12.52	10.3–16.8	11.8	5.8–18.8	0.529
	*Lactobacillaceae*	0.00	0.00–0.00	0.04	0.01–0.12	0.000
	*Porphyromonadaceae*	1.88	0.90–3.24	2.98	1.65–4.71	0.221
	*Rikenellaceae*	7.20	3.73–9.65	6.72	4.13–11.3	0.883
	*Ruminococcaceae*	23.7	16.01–30.2	23.9	14.5–27.2	0.820
	*Veillonellaceae*	5.58	4.57–14.5	6.91	3.90–14.9	0.678
**Genus**						
	*Bacteroides*	18.75	14.6–30.8	21.0	15.3–36.1	0.494
	*Coprococcus*	0.83	0.22–2.13	0.51	0.18–2.09	0.494
	*Dialister*	4.45	2.29–4.45	5.42	1.89–13.6	0.639
	*Dorea*	0.50	0.20–0.90	0.30	0.16–1.26	0.904
	*Faecalibacterium*	8.84	2.76–12.9	13.3	5.42–17.8	0.239
	*Oscillospira*	2.57	1.88–5.03	1.68	1.32–2.73	0.096
	*Parabacteroides*	1.87	0.87–3.24	2.98	1.65–4.71	0.201
	*Roseburia*	2.56	1.17–4.61	3.06	1.27–5.17	0.639
	*Ruminococcus*	1.34	0.57–3.77	0.89	0.59–1.40	0.221

**Table 7:** Quantification of lactobacilli in in vivo samples.

	Saliva			Faeces		
	Log_10_16S rRNA Copies/mL			Log_10_16S rRNA Copies/g		
	Median	IQR (25–75%)	*p* (Start–End)	Median	IQR (25–75%)	*p* (Start–End)
**Start**	7.87	0.00–9.20 *	0.548	8.28	8.05–9.46	0.014
**End**	8.54	6.73–9.28		9.71	9.18–10.4	

* Ten volunteers had concentrations below the detection limit, which were assumed to be zero during the statistical analysis.
